# Population structure, demographic history and local adaptation of the grass carp

**DOI:** 10.1186/s12864-019-5872-1

**Published:** 2019-06-07

**Authors:** Yubang Shen, Le Wang, Jianjun Fu, Xiaoyan Xu, Gen Hua Yue, Jiale Li

**Affiliations:** 1 Key Laboratory of Freshwater Aquatic Genetic Resources, Ministry of Agriculture, Shanghai Ocean University, Shanghai, 201306 China; 2 Molecular Population Genetics & Breeding Group, Temasek Life Sciences Laboratory, 1 Research Link, National University of Singapore, Singapore, 117604 Republic of Singapore; 3 Key Laboratory of Freshwater Fisheries and Germplasm Resources Utilization, Ministry of Agriculture, Freshwater Fisheries Research Center, Chinese Academy of Fishery Sciences, Wuxi, 214081 China; 4 Department of Biological Sciences, National University of Singapore, 14 Science Drive 4, Singapore, 117543 Republic of Singapore; 5 School of Biological Sciences, Nanyang Technological University, 60 Nanyang Drive, Singapore, 637551 Republic of Singapore; 6 Key Laboratory of Exploration and Utilization of Aquatic Genetic Resources, Ministry of Education, Shanghai Ocean University, Shanghai, 201306 China

**Keywords:** Grass carp, Diversity, Population structure, Evolution, Aquaculture, SNP

## Abstract

**Background:**

Genetic diversity within a species reflects population evolution, ecology, and ability to adapt. Genome-wide population surveys of both natural and introduced populations provide insights into genetic diversity, the evolutionary processes and the genetic basis underlying local adaptation. Grass carp is the most important freshwater foodfish species for food and water weed control. However, there is as yet no overall picture on genetic variations and population structure of this species, which is important for its aquaculture.

**Results:**

We used 43,310 SNPs to infer the population structure, evidence of local adaptation and sources of introduction. The overall genetic differentiation of this species was low. The native populations were differentiated into three genetic clusters, corresponding to the Yangtze, Pearl and Heilongjiang River Systems, respectively. The populations in Malaysia, India and Nepal were introduced from both the Yangtze and Pearl River Systems. Loci and genes involved in putative local selection for native locations were identified. Evidence of both positive and balancing selection was found in the introduced locations. Genes associated with loci under putative selection were involved in many biological functions. Outlier loci were grouped into clusters as genomic islands within some specific genomic regions, which likely agrees with the divergence hitchhiking scenario of divergence-with-gene-flow.

**Conclusions:**

This study, for the first time, sheds novel insights on the population differentiation of the grass carp, genetics of its strong ability in adaption to diverse environments and sources of some introduced grass carp populations. Our data also suggests that the natural populations of the grass carp have been affected by the aquaculture besides neutral and adaptive forces.

**Electronic supplementary material:**

The online version of this article (10.1186/s12864-019-5872-1) contains supplementary material, which is available to authorized users.

## Background

Genetic diversity is a central concept of evolutionary biology and molecular ecology, and plays an important role in the complexity of organisms, ecosystem recovery and the ability of a species to adapt diverse environmental conditions [1, 2]. Investigation on adaptive divergence in association with the pattern of gene flow can advance our understanding of speciation genomics [3, 4]. Adaptive divergence in response to extreme environmental variables is able to be maintained and the flanking regions can expand due to physical linkage, resulting in reduction of gene flow [3]. Thus, genetic divergence can be accumulated in the form of ‘divergence hitchhiking’ even in the background of high gene flow [5]. Alternatively, genome-wide divergence caused by selection per se can also hinder gene flow at the whole genome level, leading to further genetic divergence in the form of ‘genome hitchhiking’ [3]. Discrimination between the two patterns of ‘divergence-with-gene-flow’ can enhance our knowledge of speciation and also the underlying evolutionary forces [3, 4]. Genomic surveys of natural populations provide essential information on gene flow, genetic variation and adaptive divergence, as well as biogeographic history of species [6].

Populations with recent introduction history typically have experienced founder effects due to a small number of introduced individuals. With small effective sizes, these populations are more vulnerable to random genetic drift during the early stages of the introduction [7]. These evolutionary forces in combination with directional selection can cooperatively contribute to the decrease of genetic diversity and reduce the success of introduction [8]. Nevertheless, advantageous genetic diversity can still be maintained by a variety of selective regimes [9]. Genetic loci of higher genetic diversity under balancing selection are crucial to the survival of introduced populations during adaptation to new environments [9]. Identifying these loci and understanding the genes associated with these loci in local adaptation can help in designing novel strategies for managing biological invasions and minimizing the damage caused by non-native species [10]. In another aspect, investigation on the source populations and introduction routes of introduced populations is also valuable in understanding the evolutionary forces on adaptation in correlation to environmental factors [10]. Such information can help in designing practices for utilizing introduced resources in both breeding programs and biological control [11].

The grass carp (*Ctenopharyngodon idella*) is the most important freshwater foodfish species as well as a species for aquatic vegetation control [12], providing an excellent opportunity to study the interactions between the above evolutionary forces and genomic variations. The grass carp is native to eastern Asia and broadly distributed from the Heilongjiang River System (Amur River) southward to northern Vietnam [12]. Within its native distribution range, grass carp mainly lives in three independent river systems: Heilongjiang, Yangtze and Pearl [12]. Aquaculture of grass carp is mainly conducted within the geographical regions of the Yangtze and Pearl River Systems [13]. Its annual global production has been over five million tons since 2013 [13]. In China, the grass carp has been cultured for over 1300 years [12, 13]. Previous studies based on microsatellites showed that genetic differentiation between distant native locations was low, but statistically significant [14–17]. Further, considering the long culture history and extensive aquaculture practices, as well as the isolation by long geographic distance, the contemporary population structure of native grass carp likely has been influenced to some extent by the aquaculture. With a wide range of distribution, grass carps are under the pressure from spatially divergent selection, corresponding to heterozygous environments. Its distribution spans approximately 30 latitudinal degrees. Some environmental variables vary along latitude (e.g., extreme temperature from 0° to 38 °C) [12]. Thus, the pattern of adaptive variations at the loci associated with such environmental variables likely presents a signature of latitudinal gradient.

The grass carp has been introduced to over 80 countries for aquaculture and for controlling the water weeds [13, 18]. The earliest introduction of the grass carp was documented from Southern China to Malaysia in the 1800s [18]. However, the exact place of origin of the most introduced grass carp populations is unclear. Previous studies inferred that grass carps introduced to the USA, Europe and Japan most likely had multiple origins [16, 19]. However, no studies on tracing the origins of grass carps introduced to the other regions were reported. In comparison to the native populations, introduced grass carps in USA, Europe and Japan have reduced genetic variability and showed significant divergence [16, 19], but adapted to the new habitats very well [18]. In some species, although having experienced significant founder effects, the potential for adaptive divergence in such populations was maintained [10]. Loci showing higher genetic diversity resulting from balancing selection may play more important roles in maintaining the survival of introduced species than the loci under positive selection in the background of loss of genetic diversity [9]. However, there is no genome-wide information about the genetic variations in induced grass carp populations.

The purposes of this study were to analyze the genetic variations and to identify signatures of local selection in the native and introduced locations in the grass carp using genome-wide SNPs genotyped by using genotyping by sequencing (GBS). A total of 197 grass carp from six native and three introduced locations were genotyped with 43,310 SNPs. Our study, for the first time, sheds insights on genetic diversity, population structure, local adaption and sources of introduced grass carps, which could be useful in the aquaculture of the grass carp.

## Methods

### Sampling and data collection

A total of 197 grass carp individuals from six native and three introduced locations were collected between 2007 and 2008. The six native sampling sites were in the Heilongjiang, Yangtze and Pearl River Systems. The three sampling sites for introduced grass carp were located in Malaysia, India and Nepal, respectively (Additional file 8: Table S1 & Additional file 1: Figure S1). The annual average and peak temperatures of each sampling site were retrieved from the National Meteorological Center of China (http://www.nmc.cn) and were highly correlated (Mantel test, *R*^2^ = 0.98 and *P* < 0.001). Thus, only annual average temperature was used for further analysis (Additional file 8: Table S1). The Malaysian location was documented as being introduced in the 1800s from southern China, while the Indian location was recorded as being introduced from Hong Kong, China (the Pearl River System), in 1959 and 1968 [20]. The Nepalese location was set up by introduction from India in 1966–1967 [18]. All samples were estimated as more than one year old. Fin tissue of each fish was collected and preserved in 95% ethanol at − 20 °C. Genomic DNA was isolated using DNeasy Blood & Tissue Kit (Qiagen, Germany) and quantified using Qubit® assays (Life Technologies, USA).

### Genotyping by sequencing

Genotyping by sequencing (GBS) was conducted using the ddRAD-Seq approach [21]. Restriction enzymes PstI-HF and MspI (New England Biolabs, USA) were selected for library construction. 200 ng genomic DNA was fully digested with the two enzymes. Digested fragments were ligated with barcoded adaptors using T4-ligase (New England Biolabs, USA). Twenty four individuals were pooled for each library and the libraries were cleaned up with QIAquick PCR Purification Kit (Qiagen, Germany). The cleaned products were size selected and purified (300–500 bp) by running gels and using QIAquick Gel Extraction Kit (Qiagen, Germany), respectively. The recovered libraries were then amplified using Phusion® High-Fidelity DNA Polymerase (New England Biolabs, USA). After a final cleanup using QIAquick PCR Purification Kit (Qiagen, Germany), the libraries were sent to a NextSeq 500 platform (Illumina, USA) for 2 × 150 bp paired-end sequencing.

The program process_radtags [22] was employed to filter the raw sequencing reads with default parameters and reads with any uncalled base were removed. Clean reads were then demultiplexed and trimmed to 100 bp for in silico mapping. First, reads were mapped to the reference genome of grass carp v1.0 [23] using the program BWA-MEM with default parameters [24]. Reads with multiple targets in the reference were excluded from further analysis. Reference-aligned reads were then assembled into stacks for each individual using pstacks, implemented in the package Stacks v1.34 [22]. Six individuals randomly selected from each location were used to construct a catalogue of stacks using cstacks. Stacks from each individual were then matched against the catalogue for SNP discovery using sstacks. Finally, genotyping was conducted across all locations using the program populations implemented in Stacks with a minimum of 10× sequence depth and a success of > 70% of the individuals in each location. RAD tags were further removed if any SNP in the tag had more than two alleles and showed an observed heterozygosity of > 0.5, in order to remove paralogues [25]. As we observed that linkage disequilibrium decayed rapidly within a region of 500 bp and the linkage between SNPs from each paired-end RADtag was not significant, only the first SNP for each single-end RAD tag was retained for further analysis by populations. Hardy-Weinberg equilibrium (HWE) for each locus was examined using Genepop v4.2 [26]. Loci that deviated from HWE in a single location at the significance level of 0.01 were excluded from further analysis to filter out RAD tags, which are from polymorphic restriction sites and thus suffer from null allele.

### Analyzing population structure and phylogenetic relationship

Genetic diversity measures for each location, including observed heterozygosity (*H*_O_), expected heterozygosity (*H*_E_) and nucleotide diversity (Π), as well as *F*_ST_ value for each locus, were estimated using the program populations [22]. Population genetic divergence was estimated in the form of pairwise *F*_ST_ using the program Arlequin 3.5 [27]. Statistical significance was examined using an exact test with 10,000 permutations. Population structure at both the population and individual levels was investigated by principal component analysis using the program Eigenstrat v5.1 [28]. The pattern of population differentiation was examined in the form of isolation-by-distance (IBD) using Mantel tests with the program IBD v1.52 [29]. The genetic distance was measured as *F*_ST_/(1-*F*_ST_). For geographical distance, however, the sampling locations among different river systems are not directly connected by river courses. Considering the long distance among locations and also the complicated pattern of hydrographical connections among river systems, the pairwise geographical distance was simply modelled as linear distance between sampling localities. Population structure and admixture was investigated using a maximum likelihood method implemented in the program Admixture [30]. We carried out analysis with K (number of population units) ranging from 1 to 12 with default parameters. The K value which shows the lowest cross-validation error is suggested to be the most likely number of genetic clusters [30]. Besides using whole data set for Admixture analysis, we also used only one SNP from each paired-end RADtag in case the results were biased due to linkage between SNPs from the same paired-end RADtag. However, no difference was observed in the results between the two data sets. The phylogenetic relationship among locations was constructed using a Neighbor-Joining tree based on Nei’s standard genetic distance with the program Poptree2 [31] by bootstrapping over loci for 1000 times.

### Inferring population origins of introduced grass carp

The population origins of the introduced grass carp were inferred using an approximate Bayesian computation method [32] implemented in the program Diyabc 2.1.0 [33]. Three simple competing scenarios were designed and tested with historical demographic parameters drawn from the distribution of priors, based on the results of population structure. A total of 52 summary statistics including genic diversities, *F*_ST_ distances and Nei’s distances were estimated and used for comparing among scenarios. For the introduced location in Malaysia, two structured native locations as shown in the admixture analysis and an admixture population between the two locations were used as potential source populations to infer the ancestral population. In modeling the population origins, three competing scenarios were designed: Scenario 1, the introduced population originated from an admixture population of both river systems; Scenario 2, the introduced population originated directly from the Yangtze River System; and Scenario 3, the introduced population originated directly from the Pearl River System. These scenarios assumed no migration among populations. Two ancestral populations from the Yangtze River System and the Pearl River System, respectively, showing the closest genetic relationship with the introduced populations, were used for examination of the different scenarios. An equal number of diploid individuals were randomly selected from each of the introduced and ancestral populations. The number of individuals was determined according to the smallest population. We also tested the possibilities of applying the other native locations from each river system and found little difference between the results. The origin of the South Asian locations (India and Nepal) was inferred in a similar manner, by assuming the population in India to be the ancestral population of that in Nepal. We also tested the possibility that the population in India was founded by migrants from the population in Nepal. However, such scenarios were subsequently revealed to be less competitive.

Historical demographic parameters, including divergence time and historical population size changes that might correlate to bottleneck events for introduced populations, were considered for discrimination of the competing scenarios. We applied uniform priors and also the following parameter settings: t(n + 1) > tn > db and N > Nm; N and Nm = [10–10,000]; t1, t2 and db = [1–150]; t3 = [10–10,000]. All the other parameters were using the default settings. A total of 106 simulated datasets were generated for the three competing scenarios to produce reference tables. The scenarios and priors were pre-evaluated and at least one prior–scenario combination was designed to produce simulated datasets that are close to the observed datasets [33]. The competing scenarios were examined by posterior probabilities using a logistic regression approach with 1% of the simulated datasets. Type I and II errors were further estimated to compare the competing scenarios. The parameters were computed using the top 1% of simulated datasets which were closest to the observed datasets. The competing scenarios were examined for goodness-of-fit by conducting model checking using all calculated summary statistics with the PCA approach. Before these analyses, we filtered the SNPs by applying a missing data cut-off of 0.05 and minor allele frequency threshold of 0.05. Linkage disequilibrium between each pair of SNPs was examined as *R*^2^ with the program VCFtools [34] and SNPs with *R*^2^ < 0.05 were removed. Approximately 3000 markers were retained for estimation of each set of competing scenarios in three runs to obtain statistical robustness.

### Identifying footprints of selection

Evidence of local adaptation was detected for individual loci, using a Bayesian generalized linear mixed model involving covariance of allele frequencies and environmental variables and taking demography into account, with the program Bayenv [35]. A Bayes factor (BF) was calculated for each SNP to measure the strength of the correlation between SNP variation and environmental variables. According to the method by [35], a BF > 3 was considered as a substantial evidence for selection. The program was run for five times with an independent variance-covariance matrix of population genetic variation to achieve consistency among the runs. The genome-wide (neutral) variance-covariance matrix was estimated using the whole data set, and the mean covariance matrix over 100,000 iterations was considered. We observed that the annual average temperature was highly correlated to latitude (*R*^2^ = 0.992, *P* < 0.001). Only the loci consistently identified to be significantly associated with both temperature and latitude were retained to study latitudinal gradients.

*F*_ST_-based outlier tests were also performed to identify signatures of local selection. Firstly, a Bayesian simulation-based test implemented in BayeScan 2.1 [36] was used to identify outlier SNPs. Outliers were determined using a q-value threshold of 5%. Considering that grass carps are distributed in different river systems and that there is among-group genetic structure, a hierarchical island model [37] for identifying outlier loci was also employed using the program Arlequin 3.5 [27] with the following parameters: 50,000 simulations, 10 simulated groups, and 100 demes per group. Only outliers, with a threshold of 0.995 and a false discovery rate of 0.01, were considered as candidates under putative selection. In comparison to Bayenv, the two *F*_ST_-based outlier tests do not take demographic history into consideration. Because the null distribution of these outlier tests still does not account for the bottlenecks that have likely occurred in the introduced populations, *F*_ST_-based outlier tests were separately carried out for native locations and for all locations.

### Identifying loci and genes under putative selection

Loci under putative selection were mapped against the raw annotation of the grass carp genome. SNPs within both exons and introns were considered to have a gene feature. Coding sequences of the genes were extracted and then functionally annotated by Blast2Go [38] against the nr database with an E-value cut-off of 10–6, annotation cut-off of > 55 and a GO Weight of > 5. Enrichment of Gene Ontology (GO) terms was conducted using the program WEGO [39] with the whole gene set as background. Candidate genes were also mapped to the reference genome of zebrafish (GRCz10) using Blastn to retrieve the corresponding Ensembl gene IDs. A more detailed functional annotation of these genes was then performed by mapping to the Kyoto Encyclopedia of Genes and Genomes (KEGG) pathway database [40] using the program David with default parameters of gene count > 2 and ease = 0.1 and using all genes of zebrafish as the background [41].

### Analyzing genomic patterns of differentiation

The patterns of differentiation across the genome were examined to verify if there were genomic regions where genomic loci under putative selection formed into clusters and showed elevated divergence in comparison to the background. Estimates of genetic differentiation between the locations distributed in the Yangtze and Pearl River Systems were calculated. Fifty, 100, 150 and 200 Kb window-sized FST values were separately calculated based on the 24 longest scaffolds, ranging from 9.6 to 19.1 Mb, of grass carp reference genome using the program populations in stacks. Corresponding window-sized SNP densities were also calculated to assess if the genome-wide patterns of differentiation were independent of the patterns of SNP density.

## Results

### SNP discovery and genotyping

Genotyping by sequencing (GBS) produced an average of 10.17 million raw reads per individual. After quality control, 9.21 million reads per individual were obtained for sequence mapping and SNP discovery. A total of 458,544 RADtags were detected and 280,544 SNPs were discovered across all nine locations, with an average of 0.61 SNPs per RADtag. After removing the loci that failed to meet the filtering criteria, 43,310 SNPs were genotyped across all locations, among which 28,412 (65.6%) showed minor allele frequency (MAF) of > 0.05 across the entire data set.

### Genetic diversity and population structure

The measures of genetic diversity, including *H*_*O*_, *H*_*E*_ and *Π*, estimated based on all variant SNPs, are shown in Additional file 8: Table S1. For the six native locations, Vietnam showed slightly lower genetic diversity than the others. The genetic diversity of the introduced populations was significantly lower than that of the native locations (Mann-Whitney U test, *P* < 0.01).

Pairwise *F*_*ST*_ analysis revealed significant genetic differentiation between the native and the introduced populations with *F*_*ST*_ ranging from 0.1126 between Zhaoqing and Indian populations to 0.2399 between Vietnam and Malaysia (Additional file 9: Table S2). However, genetic differentiation between each pair of native populations was lower, with *F*_*ST*_ ranging from 0.007 between Jiujiang and Shishou to 0.052 between Hanjiang and Vietnam, but significantly different from 0 after Bonferroni corrections for multiple comparisons. Compared to the other native populations, Zhaoqing from the Pearl River System showed slightly lower genetic differentiation from the three introduced populations. The effective gene flow (Nm) estimated using *F*_ST_ ≈ 1/(4 Nm + 1) ranged from 4.604 to 12.505 among the three river systems. For native locations, genetic divergence at each individual locus was estimated between the most divergent locations (Hanjiang vs Vietnam) and also between the most distant locations (Nenjiang vs Vietnam). We observed that no loci showed *F*_ST_ > 0.5 and only < 15% of loci had *F*_ST_ > 0.1 between these sampling sites.

**Fig. 1:**
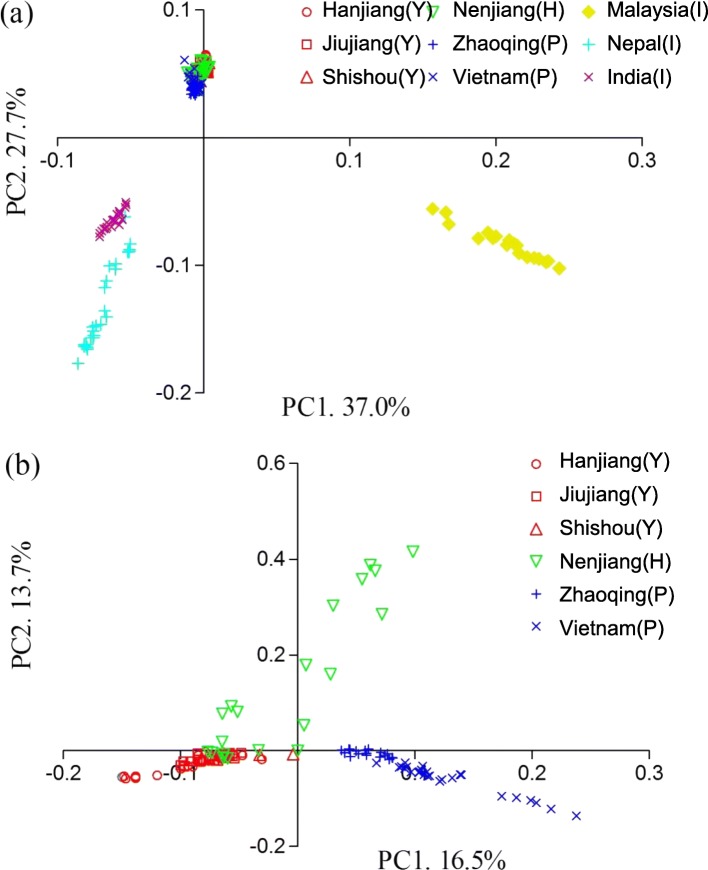
Principal component analyses for (**a**) all nine locations and (**b**) six native locations of grass carp based on all genotyped SNPs, where PC1 and PC2 contain all the meaningful signals. The native locations are annotated with the river systems where they were collected (Y, H and P corresponding to the Yangtze, Heilongjiang and Pearl River Systems, respectively), while the introduced locations are indicated with I

Principal component analysis revealed that the native locations were strikingly differentiated from the introduced locations (Fig. 1a). For the native locations, Zhaoqing and Vietnam from the Pearl River System were clearly differentiated from the locations from both the Yangtze River System (Hanjiang, Jiujiang and Shishou) and the Heilongjiang River System (Nenjiang) (Fig. 1b). Some individuals from both the Yangtze and Heilongjiang River Systems showed a very close relationship, suggesting population admixture between the two river systems (Fig. 1b). The pattern of genetic differentiation across all native locations did not show a significant signal of isolation-by-distance (*R*^2^ = 0.187, *P* = 0.16; Additional file 2: Figure S2a). Some individuals of the Heilongjiang River System are suggested to have an origin from the Yangtze River System (Fig. 1b). After excluding the Nenjiang location, we identified a strong correlation between population differentiation and geographical distance (*R*^2^ = 0.876, *P* < 0.001; Additional file 2: Figure S2b). Consistent with the above results, the neighbor-joining phylogenetic tree revealed that the native locations from the Yangtze, Heilongjiang and Pearl River Systems formed three independent clusters, respectively. The genetic distance between the introduced and the native locations was significantly longer than that among the native locations of each river system (Additional file 3: Figure S3).

**Fig. 2:**
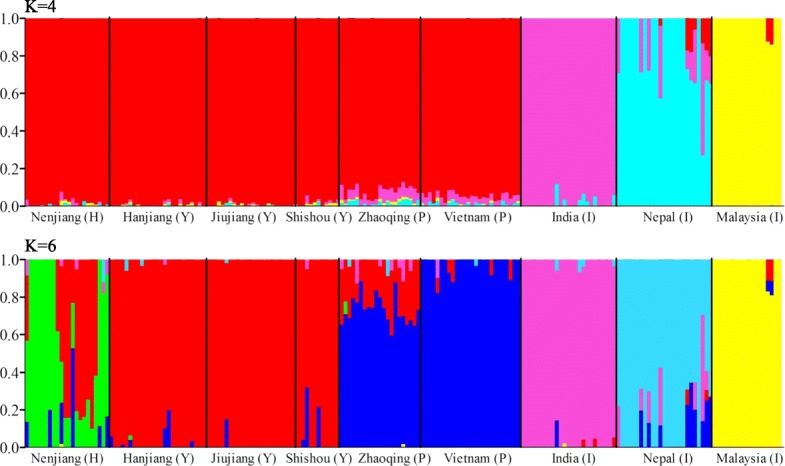
Population structure across six native locations from three river systems (Heilongjiang (H), Yangtze (Y) and Pearl (P) River Systems, respectively) and three introduced locations (I) of grass carp as inferred using Admixture at K = 4 and 6. Each vertical line represents one individual, while each colour shows the genetic composition that is assigned into a distinct genetic cluster

In Admixture analysis, the lowest cross-validation error was identified at K = 6 (CV, 0.359) followed by at K = 4 (CV, 0.360) (Additional file 4: Figure S4). For K = 4, all the native locations were grouped into one cluster, while the three introduced locations were classified into three independent clusters, respectively (Fig. 2). For K = 6, all the native locations formed into three genetic clusters corresponding to the three river systems. We also found that both Nenjiang and Zhaoqing of the Heilongjiang and the Pearl River Systems, respectively, were mixed with genetic cluster from the Yangtze River System (Fig. 2). The results for K = 5 were similar with that for K = 6, except that location India and Nepal formed into one cluster (Additional file 5: Figure S5). Consistent with the PCA results, location Nenjiang showed significant genetic composition from both the Yangtze and the Pearl River Systems. To make clear the genetic status of Nenjiang, we estimated the proportion of each genetic composition within Nenjiang at K = 6 using the program Admixture [30]. Approximately 48.8, 43.6 and 7.5% of genetic composition of Nenjiang were from the Heilongjiang, Yangtze and Pearl River Systems, respectively. Interestingly, approximately half of the individuals of Nenjiang still showed completely native genetic composition of the putative Heilongjiang River System (Additional file 5: Figure S5). To understand the effects of such admixture on the native location, we studied the pattern of population differentiation by removing the individuals (10) of Nenjiang that showed significant proportion (> 50%) of genetic composition from the other two river systems. Removal of these individuals led to Nenjiang location showing > 86.0% putative original genetic composition of Heilongjiang River System. We observed a strong signal of isolation-by-distance (*R*^2^ = 0.795, *P* < 0.001; Additional file 2: Figure S2c), consistent with the pattern of population differentiation of native locations excluding Nenjiang (Additional file 2: Figure S2b).

### Origins of the introduced grass carps

**Fig. 3:**
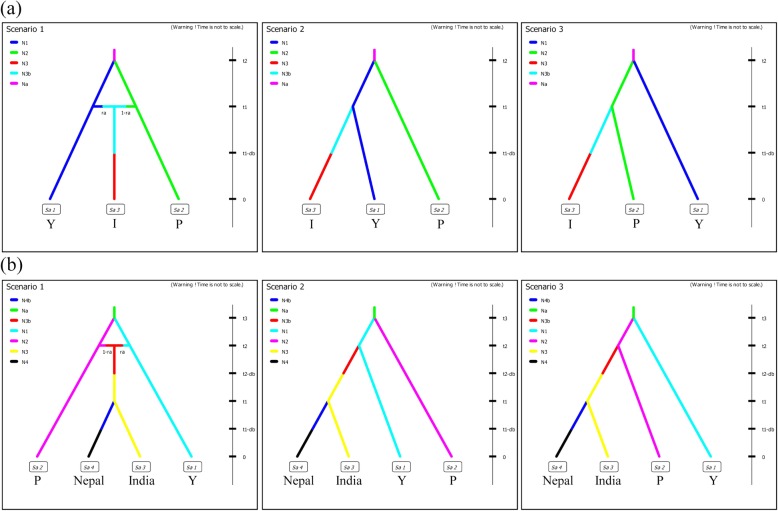
Three competing scenarios used for inferring the population origin of the introduced location in Malaysia (**a**) and the two South Asian locations in India and Nepal (**b**). For both (**a**) and (**b**), scenario 1 involves an admixture source population from the Yangtze and Pearl River Systems, while scenarios 2 and 3 have the introduced population originating from the Yangtze and the Pearl River Systems, respectively

Among the three competing scenarios, our analysis results rejected the scenarios where grass carps in Malaysia were derived from only one of the native populations. The scenario considering an admixture population, contributed by the populations of both the Yangtze and Pearl River Systems, as the most likely population source of the introduced location in Malaysia was preferred over the others (Scenario 1 in Fig. 3a and Table 1). The posterior probability of this scenario was 0.806 (95% CI: 0.782–0.829) and was much higher than the other assumed competing scenarios. Both type I and II errors were estimated to be low for this best scenario (0.021 and 0.009, respectively). With regard to the source population of India and Nepal, the scenario considering an admixture population between the populations of the Yangtze and Pearl River Systems (Scenario 1 in Fig. 3b and Table 1) was also inferred to be the most probable scenario, with the highest posterior probability of 0.927 (95% CI: 0.913–0.941). Type I and II errors for this scenario were calculated as 0.018 and 0.012, respectively. We also tested alternatives to the three competing scenarios by assuming that the introduced grass carps were derived from a ghost or an unsampled population, from each of the two river systems or an admixture population of the two river systems, but differentiated from the current populations. However, these scenarios did not change the results and showed no improvement to the current scenarios. Under the most competitive scenario, estimation of the demographic parameters revealed that both the introduced and native locations had relatively large contemporary effective population sizes (Additional file 10: Table S3). Nevertheless, all the introduced locations experienced historically significant population decline during the introduction processes (Additional file 10: Table S3).

**Table 1:** Posterior probabilities with 95% confidence interval of each of the three competing scenarios used for inferring the source population of introduced grass carp, and the type I and II errors for each competing scenario

Scenario	Posterior probability (95% CI)	Type I error	Type II error
Malaysia			
Scenario 1	**0.806 (0.782, 0.829)**	**0.021**	**0.009**
Scenario 2	0.014 (0.000, 0.110)	0.002	0.019
Scenario 3	0.180 (0.081, 0.279)	0.007	0.022
India and Nepal			
Scenario 1	**0.927 (0.913, 0.941)**	**0.018**	**0.012**
Scenario 2	0.068 (0.054, 0.082)	0.006	0.024
Scenario 3	0.005 (0.004, 0.007)	0.008	0.014

The best competing scenario is indicated in bold. Scenario 1, the introduced population originated from an admixture population of both the Yangtze and the Pearl River Systems; Scenario 2, the introduced population originated directly from the Yangtze River System; and Scenario 3, the introduced population originated directly from the Pearl River System

### Identifying loci under putative selection

Only SNPs with MAF > 0.05 were used to identify signals of selection using different approaches. Firstly, the native locations were used to infer footprints of local selection and latitudinal gradients. Using the whole data set, Bayenv identified 71 loci showing significant associations between genetic variations and latitudinal gradients (Table 2). Arlequin and BayeScan detected, in total, 276 and 183 outliers, respectively, with 128 common outliers. As revealed above, location Nenjiang was mixed with genetic composition from the other two river systems. Such admixture is likely to disturb the patterns of local selection and latitudinal gradients. Therefore, we performed another round of both correlation and outlier tests after excluding the individuals (10) in Nenjiang that showed significant genetic composition from the other river systems as stated above. Using such reduced data set, 70 loci were found to be significantly associated with latitudinal environmental variables, while 285 and 185 loci were revealed to be outliers by Arlequin and BayeScan, respectively, with 127 common outliers (Table 2). Between the two data sets, Arlequin and BayeScan detected 175 (~ 62%) and 142 (~ 77%) common loci, respectively, while no common loci with *F*_ST_-based outlier tests were identified using Bayenv.

**Table 2:** Results of Bayenv and two *F*_*ST*_-based outlier tests, Arlequin and BayeScan for identifying footprints of local selection across six native locations, using whole data set and data set reduced by excluding individuals (10) in Nenjiang showing significant genetic composition from both the Yangtze and Pearl River Systems. The common loci indicate the loci shared between two corresponding tests

Tests	Whole data set	Reduced data set	Common loci
Bayenv	71	70	0
Arlequin	276	285	175
BayeScan	183	185	142
Common *F*_*ST*_ outliers	128	127	94

To infer signatures of balancing selection that may be involved in the introduction process, all nine locations were included for *F*_ST_-based outlier tests. We also observed the same pattern as the above: that inclusion of the individuals in Nenjiang with significant genetic composition from the other two river systems produced a set of outliers slightly different to that generated after excluding these samples, both in terms of positive selection and balancing selection (Table 3). To examine if this was caused by the admixed composition of Nenjiang location or just the variations resulting from alteration of sample sets, we performed ‘leave-one-out’ tests by leaving Nenjiang in and removing any one of the other locations. We found the overall proportions of common loci in ‘leave-one-out’ tests were ~ 5% and ~ 15% higher than that of the above results generated using the data sets including and excluding the individuals of significant alien genetic compositions in Nenjiang, for Arlequin and BayeScan, respectively. It suggests the admixed composition of Nenjiang location might have slightly influenced the signature of local selection. Further considering the results of both Admixture and isolation-by-distances, the introduction of specimens to the Heilongjiang River System from the other river systems much likely occurred recently and the native gene pool of the Heilongjiang River System was still relatively independent (see discussion). The selective regimes were likely still somewhat different between migrants and natives in Nenjiang location. Therefore, only results from the reduced data set were used to study local selection for localized populations. In total, Arlequin and BayeScan identified, respectively, 335 and 191 outliers under putative positive selection, with 11 common loci, as well as 206 and 77 loci under putative balancing selection, with 74 common loci (Table 3). Considering that *F*_*ST*_-based outlier tests are vulnerable to false positives in identifying positive selection when using populations showing significant population structure and complicated demographic history, and also the significant inconsistency between the results of outlier tests in terms of positive selection (Table 3), we only took the outliers under putative balancing selection across all locations into consideration. In total, 343 and 209 loci under putative positive and balancing selection, respectively, and 70 loci associated with latitudinal gradients, were retained for further analysis. Out of these loci, four showing latitudinal variations were also detected as outliers under putative positive selection across native locations.

**Table 3:** Results of two *F*_*ST*_-based outlier tests, Arlequin and BayeScan for identifying positive and balancing selection across six native and three introduced locations, using whole data set and data set reduced by excluding individuals in Nenjiang (10) showing significant genetic composition from both the Yangtze and Pearl River Systems. The common loci indicate the loci shared between two corresponding tests

Outlier tests	Whole data set		Reduced data set		Common loci	
	Positive	Balancing	Positive	Balancing	Positive	Balancing
Arlequin	352	169	335	206	287	148
BayeScan	206	65	191	77	161	59
Common *F*_*ST*_ outliers	13	61	11	74	7	58

### Functional annotation of genes under putative selection

Mapping against the annotation of the reference genome revealed that 227 out of 618 outliers (36.7%) were associated with 210 unique genes, showing no significant deviation from the null hypothesis that 39.8% of all examined loci could hit the genomic region of a gene in the genome. A total of 154, 60 and 27 genes were revealed to be related to putative positive selection, balancing selection and latitudinal gradients, respectively (Additional file 11: Table S4). Fifty-one and 17 genes were consistently suggested to be candidates under putative positive and balancing selection, respectively, by two different *F*_ST_-based outlier tests. Out of the 618 outliers, 35 (5.7%) were detected in the coding sequences, also showing no deviation from the null hypothesis that 5.6% of all examined loci could hit the coding sequences of a gene in the genome. Fifteen (42.9%) were revealed to cause amino-acid substitutions in known proteins, within which 13 and 2 were associated with putative positive and balancing selection, respectively (Additional file 12: Table S5). This ratio was lower than that of the null hypothesis of non-synonymous mutations in coding sequences (47.4%).

GO enrichment revealed that the candidate genes were related to local adaptations covered a wide range of functions in biological processes: biological regulation, cellular process, developmental process, metabolic process, pigmentation and response to stimulus (Additional file 6: Figure S6). Genes involved in positive selection were enriched into eight KEGG pathways. The most significant pathways included ion transport (seven genes) and regulation of transcription (seven genes) (Additional file 13: Table S6). For genes under putative balancing selection, the most significant enriched KEGG pathway was calcium ion binding pathway (five genes) and hydrolase activity (five genes) (Additional file 13: Table S6). Interestingly, genes related to latitudinal gradients were mainly enriched in the integral component of membrane category, which includes 10 genes (Table 4 and Additional file 13: Table S6).

**Table 4:** Annotation of candidate genes under putative selection consistently identified by both outlier tests and Bayenv association tests

Locus	Annotation	Gene name	Overall *F*st
428	ENSDARG00000010097	Coagulation factor IXa (*F9A*)	0.288
3729	ENSDARG00000060016	Nuclear receptor binding SET domain protein 1a (*NSD1A*)	0.154
8253	ENSDARG00000023445	ATPase, Ca++ transporting, plasma membrane 3b (*ATP2B3B*)	0.194
46,475	ENSDARG00000058821	Semaphorin 5A (*SEMA5A*)	0.158
52,319	ENSDARG00000076364	Erythrocyte Membrane protein band 4.1 like 1 (*EPB41L1*)	0.187
80,064	ENSDARG00000013250	Threonyl-tRNA synthetase (*TARS*)	0.265
88,973	ENSDARG00000018418	Parathyroid hormone 1 receptor b (*PTH1RB*)	0.215
92,870	ENSDARG00000005479	Teneurin transmembrane protein 3 (*TENM3*)	0.179
113,810	ENSDARG00000074636	Centrosomal protein 170B (*CEP170B*)	0.294
128,568	ENSDARG00000073933	GRB2 associated, regulator of MAPK1-like (*GAREML*)	0.189
153,644	ENSDARG00000039241	Protein kinase C, alpha (*PRKCA*)	0.175
217,623	ENSDARG00000003403	Teneurin transmembrane protein 1 (*TENM1*)	0.223
78,588	ENSDARG00000020057	Bone morphogenetic protein receptor, type II b (*BMPR2B*)	0.089
309,269	ENSDARG00000028533	Microtubule-actin crosslinking factor 1a (*MACF1A*)	0.193
18,761	ENSDARG00000037079	Cilia and flagella associated protein 36 (*CFAP36*)	0.136
154,823	ENSDARG00000043963	si:ch211-241j12.3	0.369
149,896	ENSDARG00000052766	si:ch211-239f4.1	0.083
217,867	ENSDARG00000076998	si:ch73-92i20.1	0.207

### Genome-wide patterns of differentiation

Different window-sized *F*_ST_ values were calculated along the 24 longest scaffolds of the grass carp genome. Using a sliding window of 150 kb, some specific genomic regions were revealed to have higher than average differentiation (Additional file 7: Figure S7). Examination using different sizes of sliding window (50, 100, 200 and 250 kb) did not change the results. In order to test if the *F*_ST_ elevated regions are due to the non-random distribution of SNPs, the genome-wide patterns of *F*_*ST*_ were compared with those of SNP density for the same sliding window size. We found that the distribution patterns of *F*_ST_ were independent of those of SNP density (Additional file 7: Figure S7a and S7b). Interestingly, we observed that SNPs that were identified to be under putative selection occurred much more commonly within some specific genomic regions showing elevated differentiation, in comparison to the overall background (e.g., within the four long scaffolds, CI01000000, CI01000004, CI01000005 and CI01000030 (Additional file 7: Figure S7c)). The size of the largest ‘genomic island’ was approximately 400 Kb in scaffold CI01000005. Such specific genomic regions were also observed to be independent of SNP density.

## Discussion

In this study, we investigated range-wide population structure of native locations and the origins of introduced locations of grass carp in South and Southeast Asia, as well as local selection and genome-wide patterns of differentiation, using 43,310 SNPs covering the whole genome. This study has important implications for understanding genetic differentiation under diverse environments for freshwater fish species with a wide range of geographical distribution.

### Genetic diversity of native and introduced grass carps

In grass carp, we observed that genetic diversity in the introduced locations was significantly reduced in comparison to that in the native locations. This result is consistent with a previous study on assessment of genetic diversity in introduced locations in the USA, Europe and Japan and native locations in the Yangtze River System using mitochondrial DNA [19]. As revealed by demographic analysis, such a decline of genetic diversity is much likely caused by founder effects during population introduction as well as sequential genetic drift and directional selection. At the first stage of colonization of new habitats, populations having experienced founder events are particularly sensitive to various evolutionary forces. Genetic drift can work as the main evolutionary force, randomly fixing alleles and rapidly driving differentiation between the introduced and parental populations [8]. Meanwhile, multiple directional selections tend to fix specific alleles and can also play important roles in the process of colonization, resulting in continuous loss of genetic diversity and increase of population differentiation [42]. However, loss of genetic diversity does not necessarily indicate loss of survival potential in new habitats [43]. In our previous study, we observed that Asian seabass (*Lates calcarifer*) in Australia, which separated from the population in Southeast Asia 1.5 million years ago, lost approximately 60% of genetic diversity, but successfully adapted to and colonized the new continent [44]. Here, we observed that the contemporary effective sizes of populations in the introduced locations have recovered to a considerable level. In addition, the grass carp has been introduced to over 80 counties for aquaculture and controlling water weeds in water ways [18]. All these data suggest that the grass carps have a high capability to adapt to new habitats. Therefore, it is an ideal species for controlling water weeds and aquaculture in many countries. Certainly, to avoid issues of biological invasion, triploid grass carp should be used for controlling water weeds.

### High gene flow among native grass carp populations

Typically, freshwater fishes are more isolated by various geographical factors than marine fishes, and thus have a lower level of gene flow [45]. In this study, we observed that pairwise population genetic differentiation was very low, consistent with previous studies using microsatellites [14, 16]. Considering the geographical isolation among the three river systems, such results are rather unexpected. Since the grass carp has been cultured for food in China for more than 1300 years [12, 13], these surprising results strongly suggest that high gene flow among the three river systems is not only naturally occurring but also by the aquaculture activities. Interestingly, the observed pattern of population differentiation did not conform to isolation-by-distance across the whole data set. However, after excluding the individuals in Nenjiang location that were suggested to have recent origins from the Yangtze and Pearl River Systems, the remaining samples showed a strong signal of isolation-by-distance. This result indicates that although human-induced gene flow might have played some role in shaping the overall population structure of the grass carp, it only showed a significant effect in the Heilongjiang River System.

According to the historical records [12], grass carp were abundant in both the Yangtze River System and the Pearl River System, and were widely captured from the wild as seeds for aquaculture locally. There was no practical need to introduce grass carp between the two river systems. On the other hand, it is reasonable that gene flow can be high between the two river systems because they partially overlap in geography. For these reasons, migration occurred much more naturally, and thus the population differentiation showed a strong pattern of isolation-by-distance. However, we cannot exclude the possibility that human activities may have played an important role in the dispersal of grass carp. Such gene flow might be merely induced so randomly that it can also contribute to a pattern of isolation-by-distance. In contrast to the Yangtze and the Pearl River systems, the distribution and culture of grass carps in the Heilongjiang River System have never been abundant nor considered as a major aquaculture practice according to both historical records and current official fishery statistics [12, 13]. Geographically, the Heilongjiang River System is completely isolated from the Yangtze and Pearl River Systems. On the other hand, the Nenjiang location showed a signature of admixture from the three studied river systems. Hence, the low genetic differentiation between this river system and the other river systems indicates that human-induced dispersal played a more important role than natural gene flow. In fact, grass carps in the Heilongjiang River System grow slower than in the other river systems due to low water temperature [46]. Therefore, seeds from the other river systems, particularly from the Yangtze River System, were commonly introduced for aquaculture purposes because of geographical adjacency [12]. This is the most likely explanation for the low genetic differentiation of grass carp between the Heilongjiang River System and the other river systems. However, it is clear that the estimates of gene flow based on genetic data should be cautious because assumptions underlying the methods might not be consistent with the actual action in the nature, particularly for the populations with large spatial distribution and complicated demographic history. Future studies should be based on approaches for direct estimation of dispersal (e.g., small GPS tracker or radio transmitters and marker-assisted migration estimates), which can substantially improve the estimates of gene flow.

It also should be noted that the genetic status of Nenjiang in the Heilongjiang River System much likely has not been significantly influenced by the recent introduction of grass carp from the other river systems. Although ~ 50% of the overall genetic composition of Nenjiang was observed from the Yangtze and Pearl River Systems, some individuals still showed completely native genetic composition of the Heilongjiang River System while most of the others showed a large proportion of genetic composition from the other two river systems. Such results likely suggest that the introduced grass carps have not randomly mated with the natives in Nenjiang. Interestingly, a significant pattern of isolation-by-distance across native locations can be observed when excluding the grass carps in Nenjiang showing significant genetic composition from the other river systems. This is also the differentiation pattern among the locations within the Yangtze and Pearl River Systems. Altogether, our results suggest that the native gene pool of the Heilongjiang River System was still relatively independent and has not been significantly influenced by the immigrants from the other river systems. It may also suggest that the reproduction and hybridization capacity for the immigrants were limited in the extreme environment and due to genomic divergence although this should be experimentally examined in future studies. Above all, the current population structure should be taken into consideration when establishing conservation strategies and utilizing the natural resources for aquaculture, particularly the grass carps of Heilongjiang River System.

### Origins of the introduced grass carps

As discussed above, we observed significant genetic heterogeneity both in terms of genetic diversity and differentiation between the native and the introduced locations, as well as historical population decline in demographic models, suggesting significant founder effects in the introduced locations. It is likely that the introduction of grass carps was not initiated under planned programs or that not all the introduced fish could adapt to the new habitats. As expected, we identified significant population structure and also a clear geographical pattern of differentiation among the three river systems in a background of high gene flow, which allows tracing back the origins of the introduced locations [47]. Interestingly, the ancestral source populations of the introduced locations in Malaysia and South Asia were inferred to be an admixture of populations from the Yangtze and Pearl River Systems. This was the most well-supported among competing scenarios. It was recorded that grass carp was first introduced into Malaysia from Southern China with the large-scale migration of Chinese people in the 1800s [20]. As shown by a history study, most of the Chinese people in Southeast Asia were from Guangdong and Fujian provinces, which to some extent geographically overlap with the Pearl and Yangtze River Systems, respectively. Thus, the Malaysian location was likely founded by immigrants from both Guangdong and Fujian provinces. As for the introduced locations in India and Nepal, the source origin was also inferred as an admixture population from both the Yangtze and Pearl River Systems, although it was recorded to be from Hong Kong, a city within the Pearl River System. Hong Kong imported almost all its freshwater fish products from mainland China [48] and it has been documented that Hong Kong has a long history of importing grass carp from the regions of both of the two river systems [48, 49]. Hence, it is likely that grass carp in Hong Kong is also an admixture of populations between the two river systems.

Taken together, our data suggest that the genetic differentiation among native populations is high enough to allow tracing the origins of the recently introduced grass carp (e.g., the populations introduced to Europe and North America [13]. These results are valuable for studying the production and physiological adaptation, as well as the living environments and habitat preferences, of both native and introduced locations. Such information can be referenced to construct comprehensive introduction plans for aquaculture of the grass carp for food or for controlling water weeds in water ways in the future. It also should be noted that the source populations don’t cover the whole native distribution areas of grass carp. Thus, we can not exclude the possibility that there are some other source populations that need further evaluation.

### Local selection across native locations

It is a great challenge to discriminate local selection from neutral processes for organisms that have experienced complicated demographic history. Neutral processes can generate the same marks on genomic architecture as local adaptation does [50, 51]. In some cases, adaptive traits show a specific distribution pattern along environmental factor. If the neutral forces are coincidentally varying along some specific environmental gradients, the difficulty of disentangling the roles of adaptive driving forces of the same environmental gradients would be greatly exacerbated [52]. Grass carp is a species with a significant signature of latitudinal distribution and a differentiation pattern of isolation-by-distance. Thus, the adaptive traits might vary in parallel with the pattern of neutral processes along latitude. These evolutionary processes limited the potential to identify the signals of local adaptation [52]. Here, we used conceptually different approaches to differentiate the signatures of local adaptation from the currents of neutral evolutionary processes [51, 53].

Grass carp originated from the Yangtze River System and expanded into the Pearl and Heilongjiang River Systems during the Pleistocene and Pliocene, respectively [12]. As the Pearl and Heilongjiang River Systems cover the southernmost and the northernmost distribution ranges, respectively, such contrasting environments have likely posed strong selective pressure on the distribution of the grass carp. Such a process of natural selection provides important clues to discriminate signals of local adaptation from genome-wide patterns of isolation-by-distance. As discussed above, we found that ~ 50% of grass carps in Nenjiang likely originated from both the Yangtze and Pearl River Systems, and the recently introduced grass carps much likely have not mated randomly with the natives. Although the native gene pool of Heilongjiang River System was still relatively independent, the above-mentioned pattern of admixture is likely to weaken or even disrupt the detection of signals of selection in localized populations. Considering the potential adverse influence, only the results, after excluding the individuals in Nenjiang carrying a large proportion of genetic composition from the other river systems, were taken into consideration for further discussion.

In a strong background of isolation-by-distance for the overall population differentiation, only 409 SNPs were detected as putative candidates for local adaptation, accounting for 1.4% of the total analyzed loci. This ratio is relatively low, compared to previous studies in fish species (2.3–10%, including Atlantic salmon [54] and Chinook salmon [55] that showed weak or non-significant genome-wide patterns of isolation-by-distance. Such results likely suggest that the power to identify loci under selection is low under any high background *F*_*ST*_, even if it does not correlate with distance. Bayenv revealed that 70 SNPs were associated with latitudinal gradients, indicating clinally adaptive divergence at these loci [56]. Moreover, 343 SNPs were identified as putative *F*_*ST*_ outliers, which likely suggest diversifying selection across distinct distribution environments of grass carp. Interestingly, no overlapping loci were identified between these conceptually different tests, suggesting a complicated demographic history for the studied grass carp locations. Functional annotation and enrichment analysis showed that most of the enriched biological function categories were common between *F*_*ST*_ outliers and Bayenv results under putative selection, although there were only four shared loci between the two types of data sets. Moreover, two pathways associated with transcription factors were also enriched, implying that transcription factors also likely play important roles in driving local adaptation. One interesting finding of this study is that genes related to latitudinal gradients were mainly enriched in the integral component of membrane category, including 10 genes. Previous studies on thermal adaption in arctic and hot-springs fish species also suggested that components in membrane played an important role in evolutionary adaptation to temperature [57]. These data suggest that the 10 genes in the integral component of membrane category are important for thermal adaption. How these genes play an important role in local adaptation remains to further study by functional analysis of these genes in model organism, including zebrafish.

### Selective pressures during introduction and colonization

Identifying genetic variations during population introduction and colonization is critical to understanding the mechanism of this evolutionary process and developing strategies for genetic control of biological invasions [10]. The grass carp has successfully been introduced to and colonized new continents, including North America, Australia and Europe, and are destroying the native species in the new habitats [13]. Although phenotypic plasticity can play important roles in rapid adaptation to novel diverse environments, fluctuations in genetic variations are likely much more important for the invasive species in response to rapid changes of selective pressures [58]. For introduced populations, founder effects, random genetic drift and directional positive selection tend to reduce genetic diversity at the whole genome level [8]. Nevertheless, many adaptation-related traits are polygenic and thus the loss of variation at these loci might not influence the fitness as quickly as loss of variation at individual loci does for qualitative traits [58]. Nevertheless, founder effects, random genetic drift and directional positive selection are strong enough to drive introduced populations of small population size towards allopatric divergence [44, 59]. Such rapid population differentiation and the underlying complicated demographic history can generate confusing signals and thus generate great challenges to the present statistical approaches to identify the signatures of positive selection [36]. In this study, we found that only 11 out of 28,412 loci (0.04%) were consistently detected by both Arlequin (335) and BayeScan (191) to be under putative positive selection for the introduced locations, much likely suggesting a considerable proportion of false positives in the detected loci. This result does not necessarily suggest positive selection in the introduced grass carps due to the low effectiveness of *F*_ST_-based outlier tests in populations of complicated demographic history [36]. We also cannot exclude the possibility that genetic changes fitting the corresponding positive selections occurred prior to introduction of the studied grass carp, so that the signal of positive selection in the form of *F*_ST_ was disturbed [10]. In total, the pressure of positive selection in the introduced grass carp is still unclear and likely has been significantly underestimated.

In comparison to directionally positive selection, many more loci (74, 0.26%) were consistently identified to be under putative balancing selection by Arlequin (206) and BayeScan (77). In particular, BayeScan showed a more conservative result than Arlequin did, which is likely because the scenarios underlying BayeScan are less sensitive to the changes of effective population size and immigration rates among studied locations [36]. In populations having experienced significant founder events, genetic drift and directional selection, higher genetic diversity at specific loci is typically maintained by putative balancing selection. Genes associated with these loci are critically important for the introduced species to survive in novel environments [9]. We observed that genes under putative balancing selection were associated with many functions in grass carp as revealed by GO enrichment analysis. However, loci under putative balancing selection have not attracted sufficient attention as much as the loci under putative positive selection, because positive selection seems to be more evidently related to spatial geographic factors and quantized environmental variables [60]. Here, we found that one non-synonymous mutation in a hot shock protein family gene, DnaJ (Hsp40) homolog, subfamily B, member 9a (*DNAJB9a*), was under putative balancing selection within the introduced locations of grass carp. This gene plays an important role in disease and stress responses [61]. Maintaining a high level of genetic variation at this locus is likely critical to the survival of the species in different environments. Thus, involving a variety of selective sweeps to maintain beneficial genetic diversity, balancing selection could be just as critical as directional positive selection to the survival of isolated populations. However, these results should be taken with caution, as loci under putative selection still suffered from false positives due to low effectiveness of outlier tests. Nevertheless, we identified evidence of both positive and balancing selection on grass carp distributed in diverse environmental conditions. These evolutionary forces in combination with the environment variables should be taken into account when designing introduction strategies for both biological control and selective breeding purposes.

### Genome-wide patterns of differentiation

The ‘divergence hitchhiking’ scenario of divergence-with-gene-flow, in which specific genomic regions show elevated differentiation than average, has been testified in some fish species (e.g., stickleback, *Gasterosteus aculeatus* [62], Atlantic cod, *Gadus morhua* [63] and cichlid species [64]. Here, we observed evidence of such a ‘genomic islands’ model of divergence-with-gene-flow in the grass carp, a freshwater fish species isolated by long geographic distance but with considerable gene flow. Independent of SNP density, the clustering of outlier SNPs within the specific genomic regions with elevated *F*_*ST*_ is not likely to be artifacts resulting from the relatively small number and the uneven distribution of markers. Consistent with conclusions in cichlid, stickleback and some other fish species, the ‘genomic islands’ model might be a common pattern of divergence with frequent gene flow [3]. Different from species with high gene flow, grass carps are geographically isolated by long distance with a strong background of isolation-by-distance, and the gene flow among populations is also low. The presence of ‘genomic islands’ or divergence hitchhiking of loci under selection is likely to suggest that the pressure of natural selection can overwhelm the effects of random genetic drift, mutation, gene flow and population subdivision [4], and selection sweeps do not work on random, isolated and physically unlinked genes [44].

## Conclusions

Using population genomic approaches, we detected low but significant genetic differentiation in native locations of grass carp and disentangled the effects of environmental factors, demographic history and human activities on contemporary genetic differentiation. The level of differentiation allows tracing the global introduction history of grass carp. The applications of different approaches identified a set of loci under putative positive and balancing selection for the native and introduced locations, respectively. The latitudinal distribution of native grass carp likely has an adaptive genetic basis, although the underlying causes remain to be elucidated. Nevertheless, local adaptation has likely played important roles in shaping the contemporary population structure of grass carp. Balancing selection is also likely indispensable to the survival of grass carp during introduction so as to adapt to new habitats. Our data shed new lights on population structure, genetic diversity, sources of introduction and the strong ability of the grass carp in adapting to diverse environments, which could, in turn, facilitate its aquaculture.

## Additional files

Additional file 1: **Figure S1.** Sampling sites of six native grass carp locations distributed in the three river systems: the Heilongjiang River (H), the Yangtze River (Y) and the Pearl River (P), and three introduced locations from Malaysia (I), India (I) and Nepal (I). The native and introduced locations are denoted as black and red solid circles, respectively. Detailed sampling information is listed in Table 1. (This figure is made from Google Maps. No explicit permission is required according to the guidelines of Google Maps (https://www.google.com/permissions/geoguidelines/). (JPG 1359 kb)

Additional file 2: **Figure S2.** The overall pattern of isolation-by-distance for (a) all six native locations, (b) five native locations excluding Nenjiang and (c) all six native locations excluding individuals from Nenjiang (10) showing significant genetic composition from both the Yangtze and Pearl River Systems, examined using Mantel tests based on all genotyped SNPs. Genetic distance was estimated as FST/(1-FST), while geographical distance was the linear distance between sampling localities. (JPG 566 kb)

Additional file 3: **Figure S3.** A phylogenetic tree showing relationships among all nine locations of grass carp, which was constructed using the Neighbour-Joining approach with bootstrap values over loci for 1000 times. (JPG 409 kb)

Additional file 4: **Figure S4.** Plot for cross-validation errors at each K value for nine grass carp locations. (JPG 357 kb)

Additional file 5: **Figure S5.** Population structure across nine locations of grass carp as inferred using Admixture at K = 5. (JPG 80 kb)

Additional file 6: **Figure S6.** Gene ontology annotations of the candidate genes under putative selection identified by outlier tests and Bayenv association tests in grass carp. Three categories: Cellular Component, Molecular Function and Biological Process, were used to visualize the potential functions of enriched genes. (JPG 157 kb)

Additional file 7: **Figure S7.** Genome-wide pattern of differentiation (a) and SNP density (b) calculated using 150 Kb sliding window size. Distribution of SNPs under putative positive selection in four scaffolds of the reference genome with the greatest number of outlier loci are shown in (c), where clusters of SNPs under putative positive selection are in shadow. (JPG 1626 kb)

Additional file 8: **Table S1.** Sampling information of six native and three introduced locations of grass carp including river systems of origin, numbers of samples, sampling localities and dates, and the annual average temperature of each sampling locality. Measures of genetic diversity including observed heterozygosity (*H*_*O*_), expected heterozygosity (*H*_*E*_) and nucleotide diversity (*Π*) are also indicated. (DOCX 14 kb)

Additional file 9: **Table S2.** Pairwise *F*_*ST*_ values among each pair of locations of grass carp. Genetic differentiation that was non-significant after Bonferroni corrections (*P* < 0.001) is denoted in bold. (DOCX 14 kb)

Additional file 10: **Table S3.** Parameters used for Diyabc modelling and its distribution for historical demographic parameters of the best competing scenario for two datasets. (DOCX 16 kb)

Additional file 11: **Table S4.** List of annotated genes under putative selection identified by outlier tests and Bayenv association tests. (XLSX 20 kb)

Additional file 12: **Table S5.** Summary of the SNPs associated with local selection that are identified to be located in coding sequences of genes and showing non-synonymous mutations. Codon variant positions with the two alternative nucleotides and the corresponding amino acid change are shown in the “Codon” and “Amino acid” columns, respectively. (DOCX 17 kb)

Additional file 13: **Table S6.** Enriched KEGG pathways and the gene counts under putative selection identified by outlier tests and Bayenv association tests. (XLSX 10 kb)

## Data Availability

Raw sequencing reads for this study are available at DDBJ database with BioProject accession no. PRJDB4785. SNP data set can be accessed via email to the corresponding authors.

## Abbreviations

BF: Bayes factor
ddRAD-seq: double digest restriction-site associated DNA
GBS: genotyping by sequencing
GO: gene ontology
HWE: Hardy-Weinberg equilibrium
IBD: isolation-by-distance
KEGG: Kyoto Encyclopedia of Genes and Genomes
MAF: minor allele frequency
PCA: principal component analysis
