# Supplementary figures and images for: Population structure, demographic history and local adaptation of the grass carp
Source: BMC Genomics. 2019 Jun 7;20:467. doi: 10.1186/s12864-019-5872-1 (PMC6555922; doi:10.1186/s12864-019-5872-1)

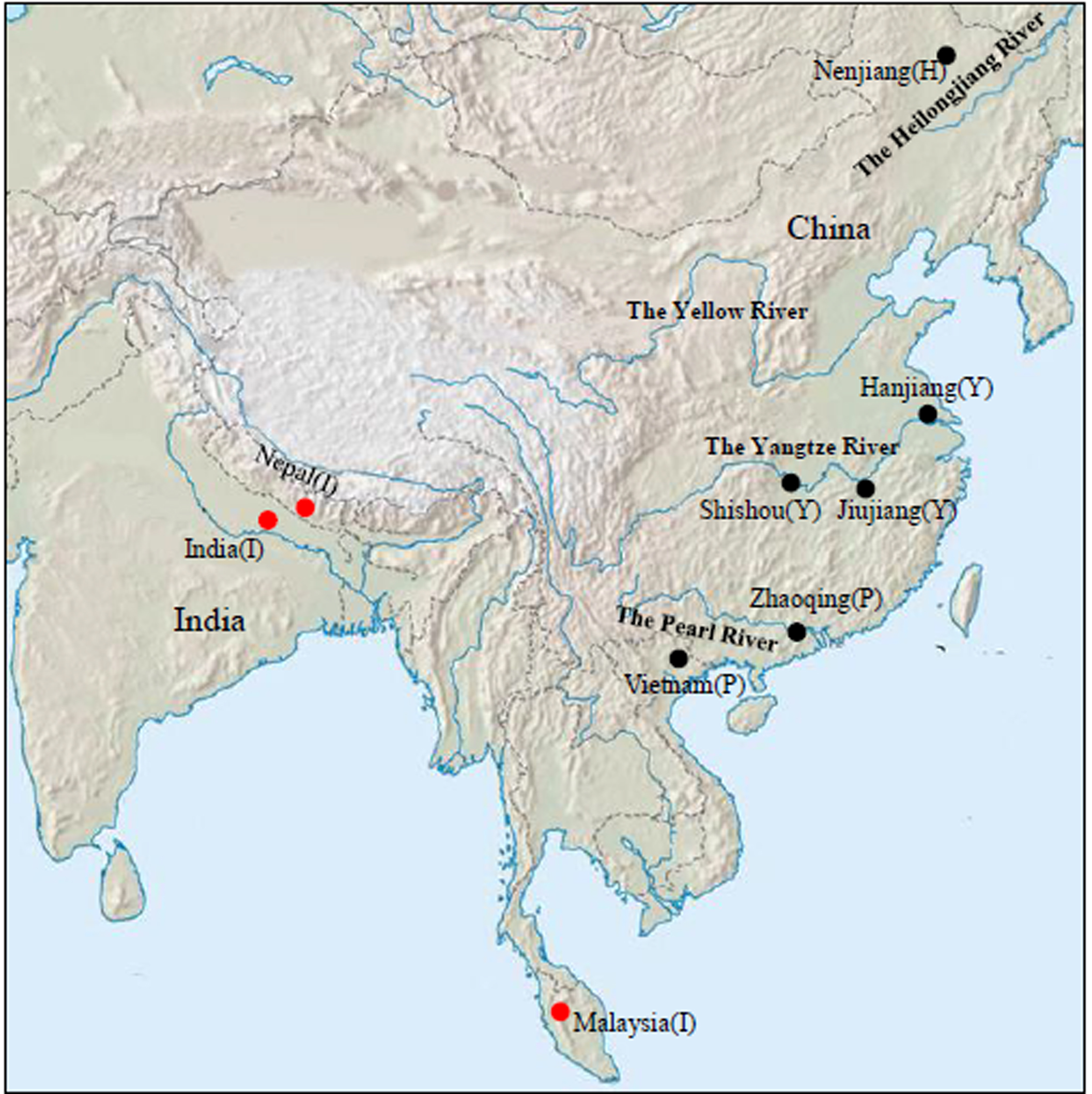

Supplement: Supplementary file 1 — Figure S1. Sampling sites of six native grass carp locations distributed in the three river systems: the Heilongjiang River (H), the Yangtze River (Y) and the Pearl River (P), and three introduced locations from Malaysia (I), India (I) and Nepal (I). The native and introduced locations are denoted as black and red solid circles, respectively. Detailed sampling information is listed in Table 1. (This figure is made from Google Maps. No explicit permission is required according to the guidelines of Google Maps (https://www.google.com/permissions/geoguidelines/). (JPG 1359 kb) [file 12864_2019_5872_MOESM1_ESM.jpg]

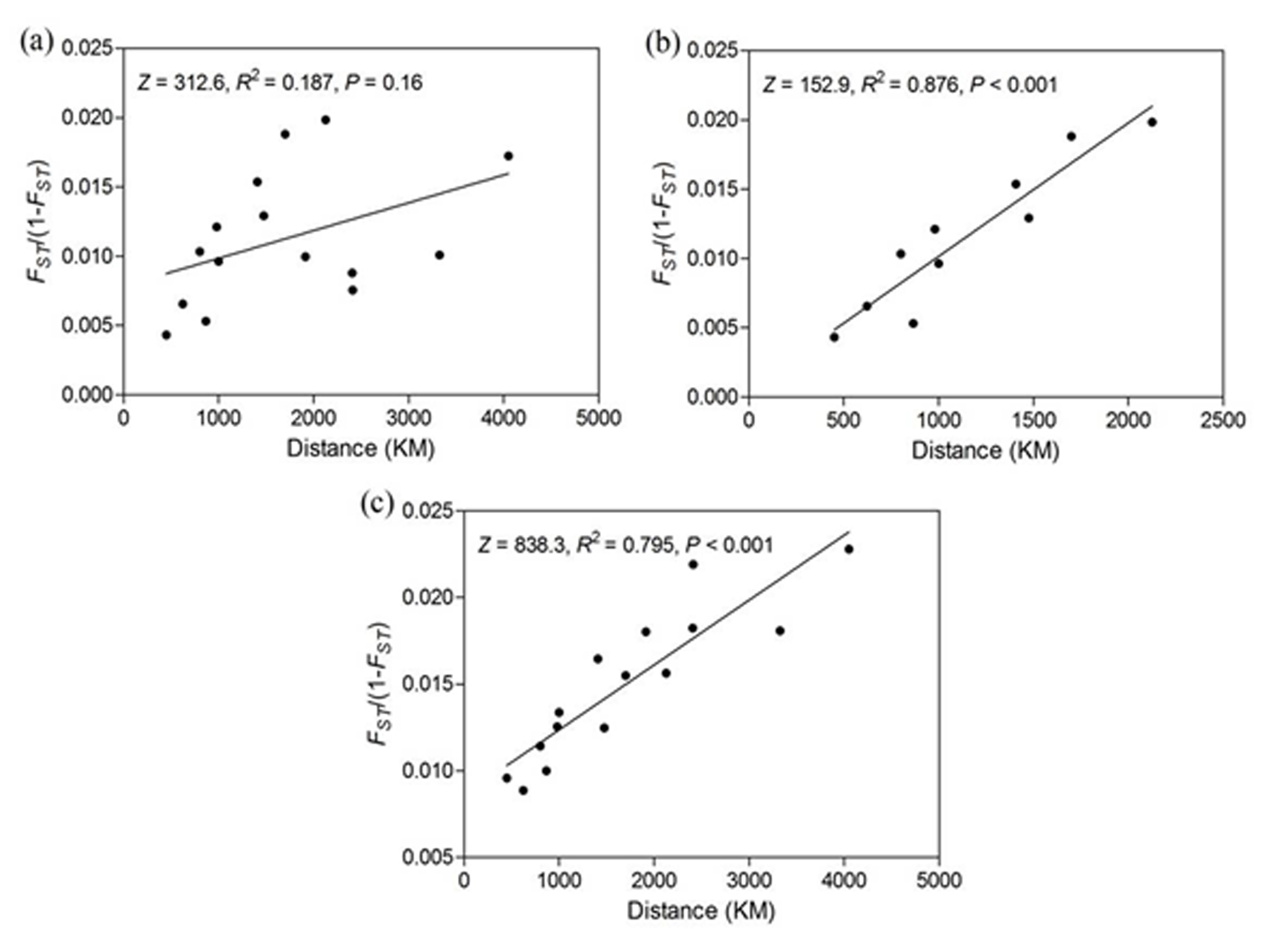

Supplement: Supplementary file 2 — Figure S2. The overall pattern of isolation-by-distance for (a) all six native locations, (b) five native locations excluding Nenjiang and (c) all six native locations excluding individuals from Nenjiang (10) showing significant genetic composition from both the Yangtze and Pearl River Systems, examined using Mantel tests based on all genotyped SNPs. Genetic distance was estimated as FST/(1-FST), while geographical distance was the linear distance between sampling localities. (JPG 566 kb) [file 12864_2019_5872_MOESM2_ESM.jpg]

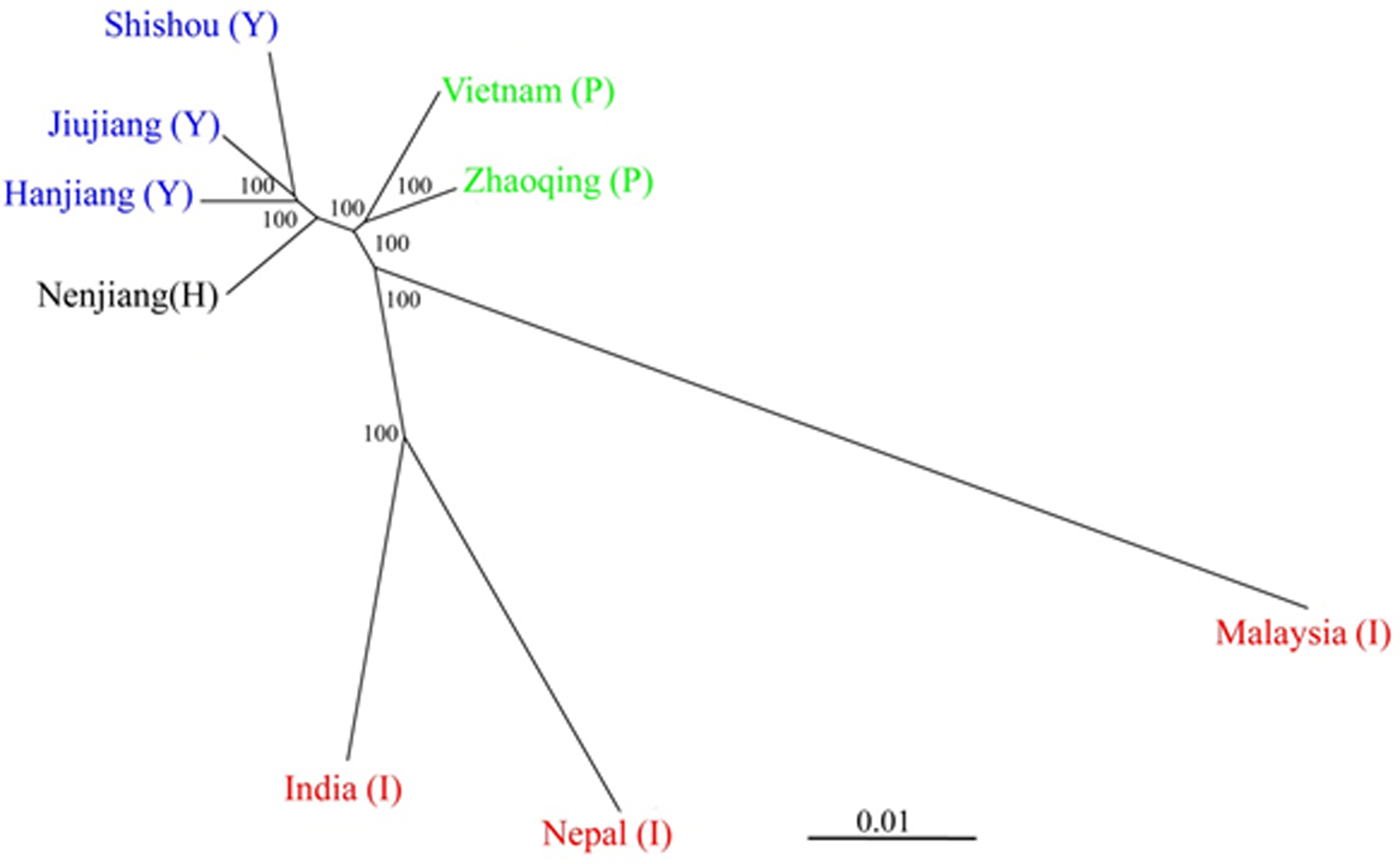

Supplement: Supplementary file 3 — Figure S3. A phylogenetic tree showing relationships among all nine locations of grass carp, which was constructed using the Neighbour-Joining approach with bootstrap values over loci for 1000 times. (JPG 409 kb) [file 12864_2019_5872_MOESM3_ESM.jpg]

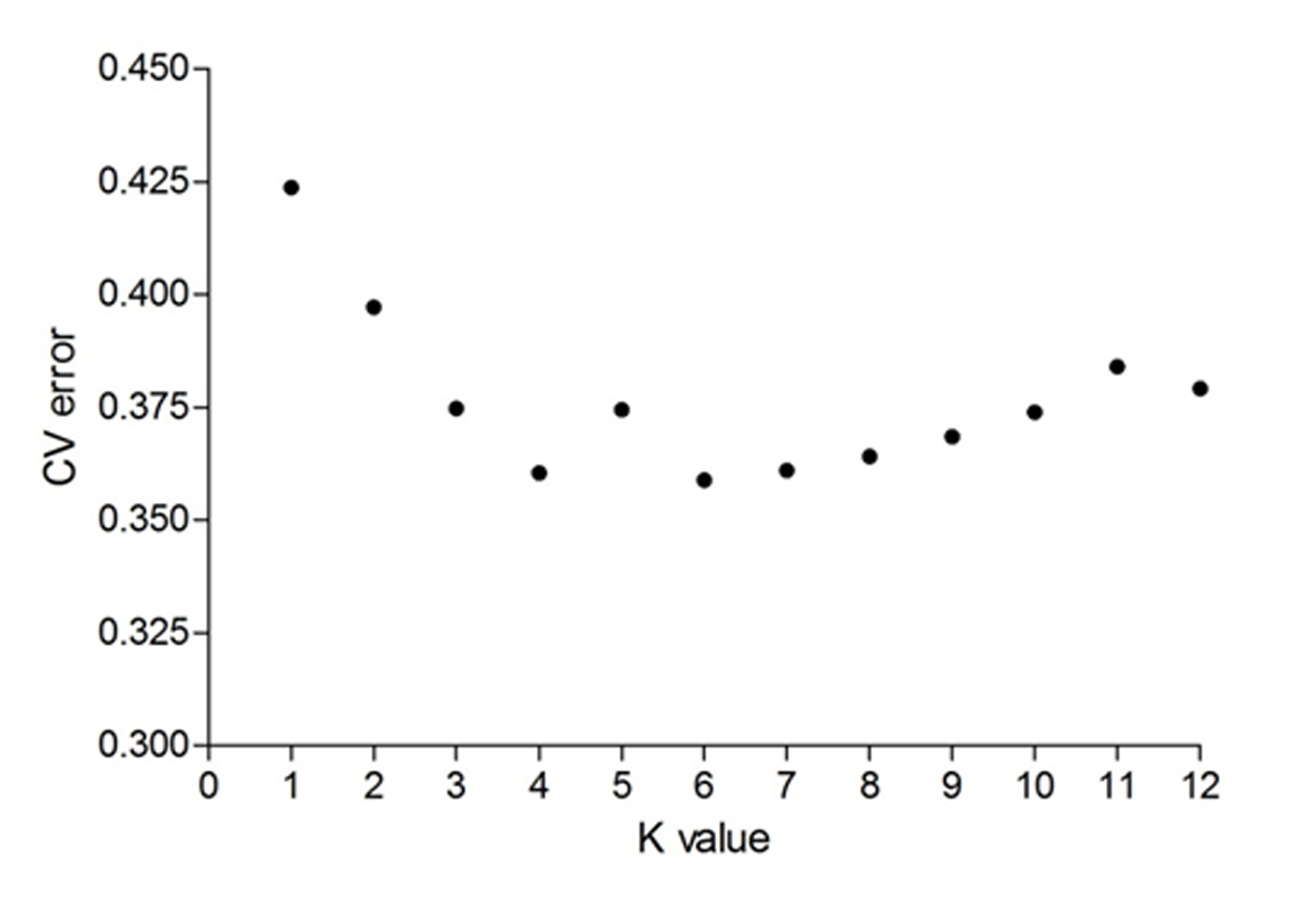

Supplement: Supplementary file 4 — Figure S4. Plot for cross-validation errors at each K value for nine grass carp locations. (JPG 357 kb) [file 12864_2019_5872_MOESM4_ESM.jpg]

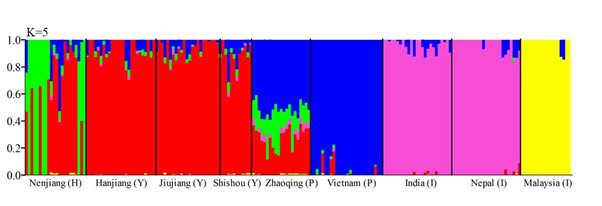

Supplement: Supplementary file 5 — Figure S5. Population structure across nine locations of grass carp as inferred using Admixture at K = 5. (JPG 80 kb) [file 12864_2019_5872_MOESM5_ESM.jpg]

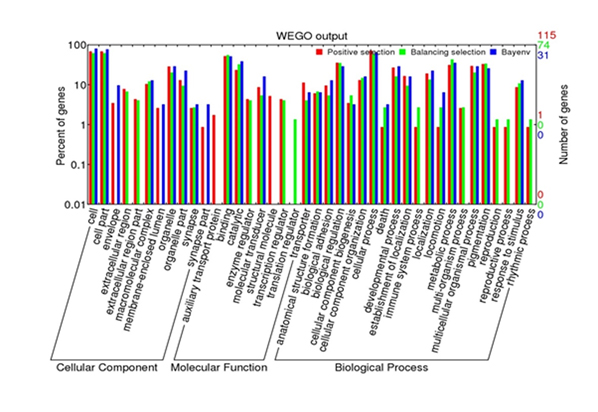

Supplement: Supplementary file 6 — Figure S6. Gene ontology annotations of the candidate genes under putative selection identified by outlier tests and Bayenv association tests in grass carp. Three categories: Cellular Component, Molecular Function and Biological Process, were used to visualize the potential functions of enriched genes. (JPG 157 kb) [file 12864_2019_5872_MOESM6_ESM.jpg]

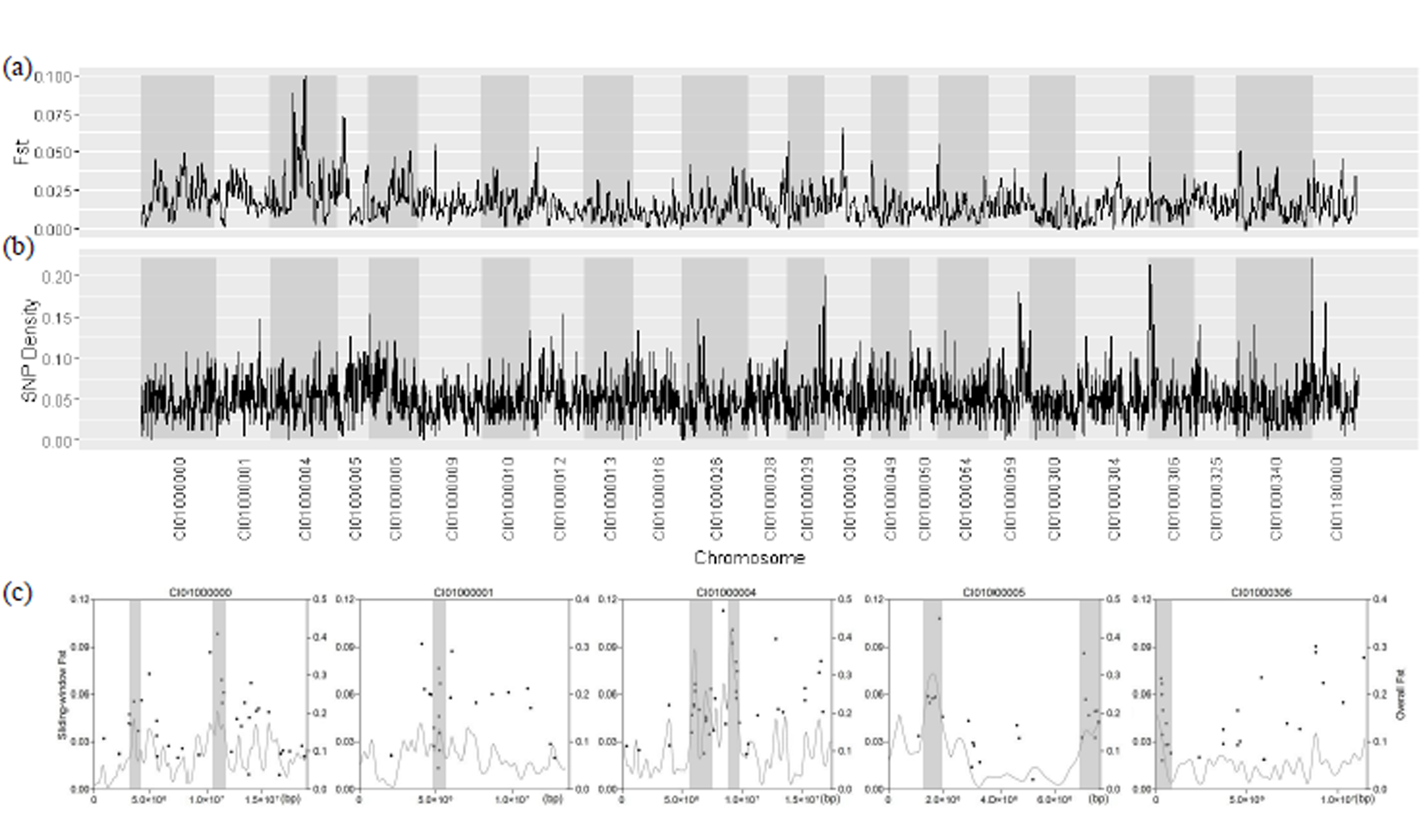

Supplement: Supplementary file 7 — Figure S7. Genome-wide pattern of differentiation (a) and SNP density (b) calculated using 150 Kb sliding window size. Distribution of SNPs under putative positive selection in four scaffolds of the reference genome with the greatest number of outlier loci are shown in (c), where clusters of SNPs under putative positive selection are in shadow. (JPG 1626 kb) [file 12864_2019_5872_MOESM7_ESM.jpg]
