# Supplementary material for: Population structure, demographic history and local adaptation of the grass carp
Source: BMC Genomics. 2019 Jun 7;20:467. doi: 10.1186/s12864-019-5872-1 (PMC6555922; doi:10.1186/s12864-019-5872-1)
Supplement: Supplementary file 8 — Table S1. Sampling information of six native and three introduced locations of grass carp including river systems of origin, numbers of samples, sampling localities and dates, and the annual average temperature of each sampling locality. Measures of genetic diversity including observed heterozygosity (HO), expected heterozygosity (HE) and nucleotide diversity (Π) are also indicated. (DOCX 14 kb) [file 12864_2019_5872_MOESM8_ESM.docx]

**Table S1** Sampling information of six native and three introduced locations of grass carp including river systems of origin, numbers of samples, sampling localities and dates, and the annual average temperature of each sampling locality. Measures of genetic diversity including observed heterozygosity (*H_O_*), expected heterozygosity (*H_E_*) and nucleotide diversity (*Π*) are also indicated.

| Samples | Origin | N | Longitude | Latitude | Date | Temperature | *H_O_* | *H_E_* | *Π* |
| --- | --- | --- | --- | --- | --- | --- | --- | --- | --- |
| Nenjiang | Heilongjiang River System | 22 | 125.22 | 49.21 | 2007 | 4.3 | 0.197 | 0.202 | 0.207 |
| Hanjiang | Yangtze River System | 26 | 119.43 | 32.35 | 2007 | 15.4 | 0.200 | 0.201 | 0.205 |
| Jiujiang | Yangtze River System | 23 | 115.96 | 29.72 | 2007 | 17.7 | 0.202 | 0.205 | 0.210 |
| Shishou | Yangtze River System | 11 | 112.39 | 29.74 | 2007 | 16.9 | 0.210 | 0.204 | 0.214 |
| Zhaoqing | Pearl River System | 21 | 112.53 | 23.08 | 2007 | 22.7 | 0.203 | 0.201 | 0.206 |
| Vietnam | Pearl River System | 26 | 105.98 | 21.12 | 2008 | 24.8 | 0.190 | 0.191 | 0.195 |
| Malaysia | Introduced | 18 | 101.15 | 4.58 | 2008 | Na. | 0.136 | 0.126 | 0.130 |
| India | Introduced | 25 | 83.37 | 26.76 | 2008 | Na. | 0.171 | 0.168 | 0.171 |
| Nepal | Introduced | 25 | 85.03 | 27.42 | 2008 | Na. | 0.161 | 0.152 | 0.155 |
