# Supplementary material for: Population structure, demographic history and local adaptation of the grass carp
Source: BMC Genomics. 2019 Jun 7;20:467. doi: 10.1186/s12864-019-5872-1 (PMC6555922; doi:10.1186/s12864-019-5872-1)
Supplement: Supplementary file 9 — Table S2. Pairwise FST values among each pair of locations of grass carp. Genetic differentiation that was non-significant after Bonferroni corrections (P < 0.001) is denoted in bold. (DOCX 14 kb) [file 12864_2019_5872_MOESM9_ESM.docx]

**Table S2** Pairwise *F_ST_* values among each pair of locations of grass carp. Genetic differentiation that was non-significant after Bonferroni corrections (*P* < 0.001) is denoted in bold.

|  | Nenjiang | Hanjiang | Jiujiang | Shishou | Zhaoqing | Vietnam | India | Nepal | Malaysia |
| --- | --- | --- | --- | --- | --- | --- | --- | --- | --- |
| Nenjiang | - |  |  |  |  |  |  |  |  |
| Hanjiang | 0.0260 | - |  |  |  |  |  |  |  |
| Jiujiang | 0.0228 | 0.0154 | - |  |  |  |  |  |  |
| Shishou | 0.0196 | 0.0123 | **0.0073** | - |  |  |  |  |  |
| Zhaoqing | 0.0272 | 0.0351 | 0.0337 | 0.0279 | - |  |  |  |  |
| Vietnam | 0.0448 | 0.0515 | 0.0484 | 0.0449 | 0.0238 | - |  |  |  |
| India | 0.1265 | 0.1307 | 0.1282 | 0.1289 | 0.1126 | 0.1297 | - |  |  |
| Nepal | 0.1540 | 0.1580 | 0.1532 | 0.1596 | 0.1384 | 0.1592 | 0.184 | - |  |
| Malaysia | 0.2295 | 0.2255 | 0.2224 | 0.2336 | 0.2205 | 0.2399 | 0.317 | 0.3501 | - |
