# Supplementary material for: Population structure, demographic history and local adaptation of the grass carp
Source: BMC Genomics. 2019 Jun 7;20:467. doi: 10.1186/s12864-019-5872-1 (PMC6555922; doi:10.1186/s12864-019-5872-1)
Supplement: Supplementary file 10 — Table S3. Parameters used for Diyabc modelling and its distribution for historical demographic parameters of the best competing scenario for two datasets. (DOCX 16 kb) [file 12864_2019_5872_MOESM10_ESM.docx]

**Table S3** Parameters used for Diyabc modelling and its distribution for historical demographic parameters of the best competing scenario for two datasets.

| Parameter | For Malaysia | | | | |  | For India and Nepal | | |
| --- | --- | --- | --- | --- | --- | --- | --- | --- | --- |
|  | mean | q050 | q950 |  | | | mean | q050 | q950 |
| N1 | 9060.00 | 7110.00 | 9950.00 |  | 6450.00 | | | 3760.00 | 8890.00 |
| N2 | 7580.00 | 4510.00 | 9710.00 |  | 3970.00 | | | 1590.00 | 7200.00 |
| N3 | 8710.00 | 4810.00 | 9970.00 |  | 9130.00 | | | 6860.00 | 9980.00 |
| N4 | n.a. | n.a. | n.a. |  | 4170.00 | | | 412.00 | 9170.00 |
| t1 | 97.90 | 92.00 | 100.00 |  | 84.70 | | | 64.90 | 96.60 |
| t1-db | 31.20 | 13.80 | 53.90 |  | 89.70 | | | 75.50 | 97.80 |
| N4b | n.a. | n.a. | n.a. |  | 451.00 | | | 127.00 | 933.00 |
| t2 | 232.00 | 103.00 | 379.00 |  | 94.50 | | | 81.50 | 100.00 |
| t2-db | n.a. | n.a. | n.a. |  | 32.20 | | | 10.40 | 69.40 |
| N3b | 34.00 | 15.70 | 58.10 |  | 575.00 | | | 205.00 | 1150.00 |
| ra | 0.11 | 0.03 | 0.23 |  | 0.48 | | | 0.26 | 0.70 |
| t3 | n.a. | n.a. | n.a. |  | 195.00 | | | 94.70 | 318.00 |
| Na | 9880.00 | 9590.00 | 10000.00 |  | 9130.00 | | | 7490.00 | 9950.00 |
