# Supplementary material for: Population structure, demographic history and local adaptation of the grass carp
Source: BMC Genomics. 2019 Jun 7;20:467. doi: 10.1186/s12864-019-5872-1 (PMC6555922; doi:10.1186/s12864-019-5872-1)
Supplement: Supplementary file 12 — Table S5. Summary of the SNPs associated with local selection that are identified to be located in coding sequences of genes and showing non-synonymous mutations. Codon variant positions with the two alternative nucleotides and the corresponding amino acid change are shown in the “Codon” and “Amino acid” columns, respectively. (DOCX 17 kb) [file 12864_2019_5872_MOESM12_ESM.docx]

**Table S5** Summary of the SNPs associated with local selection that are identified to be located in coding sequences of genes and showing non-synonymous mutations. Codon variant positions with the two alternative nucleotides and the corresponding amino acid change are shown in the “Codon” and “Amino acid” columns, respectively.

| SNP ID | Scaffold | Position | Codon | Amino acid | Gene name | Outlier tests |
| --- | --- | --- | --- | --- | --- | --- |
| *Positive selection* | |  |  |  |  |  |
| 99155 | CI01000052 | 3473993 | G>A | E/K | Pim proto-oncogene, | Bayenv |
|  |  |  |  |  | serine/threonine kinase, |  |
|  |  |  |  |  | related 56 |  |
| 98085 | CI01000051 | 7545874 | G>C | E/D | Arrestin domain | Arlequin |
|  |  |  |  |  | containing 2 |  |
| 90450 | CI01000047 | 7139072 | G>A | R/H | Myocardin | Arlequin |
| 89207 | CI01000047 | 3237850 | G>C | M/I | Pleckstrin homology domain | Arlequin |
|  |  |  |  |  | containing, family M member 1 |  |
| 86 | CI01000000 | 10283430 | C>T | P/L | Neuronal PAS domain protein 4a | Arlequin/BayeScan |
| 80148 | CI01000039 | 3687813 | A>G | I/V | Methylcrotonoyl-CoA | Arlequin |
|  |  |  |  |  | carboxylase 2 (beta) |  |
| 34763 | CI01000012 | 7820542 | G>T | A/S | Matrilin 3b | Bayenv |
| 260956 | CI01180000 | 6764835 | C>T | R/C | MAP/microtubule affinity- | Bayenv |
|  |  |  |  |  | regulating kinase 3a |  |
| 221988 | CI01000340 | 6250948 | G>T | C/F | Nuclear autoantigenic sperm | BayeScan |
|  |  |  |  |  | protein (histone-binding) |  |
| 195648 | CI01000306 | 549004 | G>A | E/K | Meningioma expressed | Arlequin |
|  |  |  |  |  | antigen 5 (hyaluronidase) like |  |
| 164607 | CI01000166 | 396870 | C>T | R/W | Solute carrier family 24 | Arlequin |
|  |  |  |  |  | (sodium/potassium/calcium |  |
|  |  |  |  |  | exchanger), member 5 |  |
| 130005 | CI01000080 | 683346 | A>C | Q/P | Thrombospondin 4a | Arlequin |
| 128588 | CI01000080 | 1097230 | T>C | C/R | Protein prenyltransferase alpha | Arlequin |
|  |  |  |  |  | subunit repeat containing 1 |  |
| *Balancing selection* | |  |  |  |  |  |
| 156027 | CI01000139 | 145189 | G>C | W/C | Cytoplasmic polyadenylation | Arlequin |
|  |  |  |  |  | element binding protein 1a |  |
| 106698 | CI01000055 | 6511021 | C>T | T/I | DnaJ (Hsp40) homolog, | BayeScan |
|  |  |  |  |  | subfamily B, member 9a |  |
